# Dislocation of a McMinn-Like Prosthesis with Distinctive Metallosis and Fracture of the Os Ilium

**DOI:** 10.1155/2021/6151679

**Published:** 2021-11-10

**Authors:** Peter Caspar Bünemann, Stefan Luck, Malte Ohlmeier, Thorsten Gehrke, Tobias Malte Ballhause

**Affiliations:** Department of Orthopedic Surgery, ENDO-Clinic Hamburg, Holstenstr. 2, 22767 Hamburg, Germany

## Abstract

Osteoarthritis of the hip joint (coxarthrosis) is the most common hip disease in adults. Since the 1960s, total hip arthroplasty (THA) has made great progress and is nowadays one of the most frequently used procedures in orthopedic surgery. Different bearing concepts exist in various implant designs. A metal-on-metal bearing can create metal debris and lead to metallosis. We present a unique case of a 78-year old woman, who received hip resurfacing with a McMinn-like prosthesis 15 years ago. Over the cause of time, metallosis developed and created a bone cyst in the Os ilium, and osteolysis led to a dislocation of the femoral implant. A minor stumble fall led to a fracture of the Os ilium. We present our treatment method with implantation of a cemented THA and refill of the bone cyst with bone from allogene femoral heads. The surgery led to a reconstruction of the physiological center of rotation in the hip. Consequently, to the inpatient stay, the patient took part in a follow-up treatment with intensive physiotherapy. Taken together, the results after total hip arthroplasty are more superior to other hip surface replacements in terms of longevity and patient satisfaction. Especially, metal-on-metal bearing couples carry a great risk of metallosis, which goes a long with pseudotumors, osteolysis, and the elevated metal ions in the blood. Since this case is unique in the literature, no guidelines are noted for surgical treatment. In our opinion, a periprosthetic fracture of this type should be performed in a hospital using extensive endoprosthetic expertise.

## 1. Introduction

Osteoarthritis of the hip joint (coxarthrosis) is the most common hip disease in adults. A primitive coxarthrosis or idiopathic coxarthrosis is observed when no anatomical abnormality in the hip joint is noted. Degenerative changes of the articular cartilage are the major cause of joint destruction [1]. Secondary coxarthrosis occurs with dysplasia of the hip joint. In addition, the unfavorable joint anatomy is the most frequent cause of secondary coxarthrosis, in which osteoarthritis of the hip is posttraumatic [2].

Sir John Charnley performed the first modern total hip arthroplasty (THA) in the 1960s [3]. Since then, THA has offered remarkable long-term results, and it is one of the most frequently used clinical procedures in orthopedic surgery. In 2019, a total of 157,681 THA surgical procedures were performed in Germany [4]. However, the figure is expected to rise in the coming years owing to an increase in life expectancy and demand for mobility. The THA average 10-year survival rate is 93%-97%. However, the 25-year implant survival rate is 60-80% [5].

Despite excellent results, significant limitations in functional activity after THA are still observed. Thus, bone-sparing hip resurfacing was developed as a possible alternative to total resection of the femoral head and neck. Hip resurfacing was a bone-saving surgery, especially for younger patients. A metal-on-metal bearing was more stable in hip resurfacing when compared to a metal-on-polyethylene or ceramic-on-polyethylene bearing on ordinary THA [6].

Every bearing surface shows abrasive wear. The debris from metal-on-metal bearings leads to local tissue damage and systemic reactions. Local tissue damage, such as metallosis and cystic or solid pseudotumor, has increased concerns about metal-on-metal bearing [7]. We present a unique case, in which severe metallosis with pelvic cysts lead to a consequential Os ilium fracture.

## 2. Case Presentation

We report a case of a 78-year-old Caucasian female patient with an ileum fracture and pronounced acetabular cysts after THA was performed with an McMinn-like prosthesis. The patient developed secondary coxarthrosis because of dysplasia of the hip. McMinn-like prosthesis was implanted in a University Clinic in 2004 (Birmingham Hip Resurfacing: Acetabular Cup 50 mm Diameter HAP Coated, Femoral Head 42 mm Diameter cemented, Finsbury Orthopaedics, Leatherhead, UK). For further dysplasia coxarthrosis, the endoprosthetic restoration was conducted in 2007 on the right side, using an McMinn-like prosthesis. After the operation, the patient was symptom-free for many years with regular mobility. Radiological follow-up reveals a large acetabular cyst on the left side, monitored over the years without any intervention.

Furthermore, the patient underwent a spinal fusion in the lumbar spine area in 2011. The patient's secondary diagnoses are substituted hypothyroidism, arterial hypertension, psoriasis vulgaris, moderate tricuspid valve insufficiency, chronic lymphocytic leukemia, and the status after breast cancer in 2001 at the left side, treated with mastectomy and chemotherapy.

**Figure 1:**
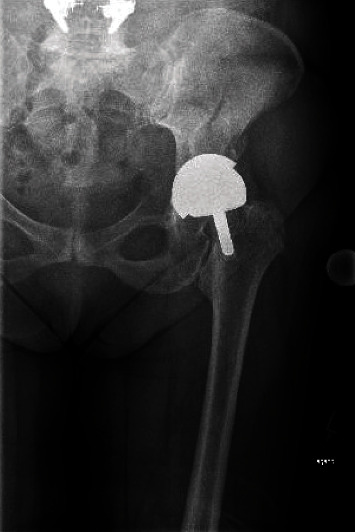
Initial X-ray of the fracture. After a stumble fall, the patient presented herself to an orthopedist. An anteroposterior X-ray of the pelvis was conducted and showed a fracture of the pelvis with a dislocation of the prosthesis.

**Figure 2:**
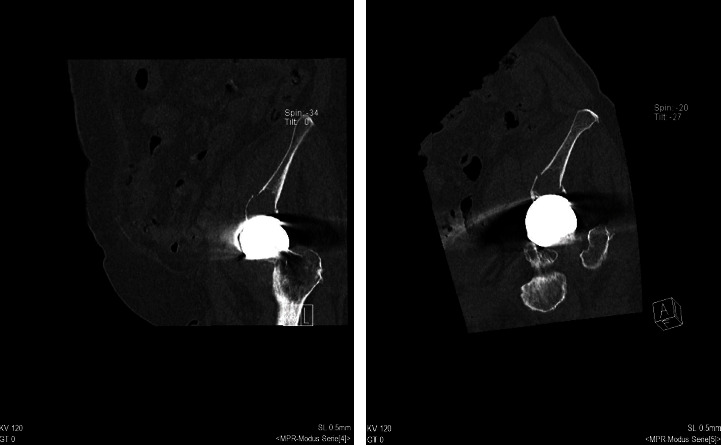
Preoperative CT of the pelvis. (a) shows the large osteolysis in the sagittal plane. (b) is the corresponding coronal plane of the patient's left hip.

The patient contacted our outpatient clinic because she had tripped over the stairs in her house. Afterward, she had constant and severe pain in the hip. The resident doctor conducted computed tomography (CT) and native x-rays on the hip to reveal the affected part (Figures 1 and 2). The CT showed the full extent of an acetabular fracture and the extension to the iliac bone. Since the fall, the patient has been immobilized on two forearm walking aids and dependent on regular painkillers, taking paracetamol 3 grams a day.

The outcome of the clinical examination shows that the patient (162 cm, 58 kg, body mass index 22.1) has a limping gait pattern. In the left hip, the surgery was performed via a posterior approach to the hip. The scar tissue and the soft tissue were irritation-free. Mobility of the left hip was for extension/flexion: 0-0-90, abduction/adduction: 20-0-10, and external rotation/internal rotation: 20-0-10. Pronounced pain with terminal flexion, adduction, and internal rotation is also observed. Mobility of the right hip for extension/flexion: 0-0-110, abduction/adduction: 25-0-15, and external rotation/internal rotation: 30-0-10 is noted. The Lasègue sign was negative. No spinal tapping pain is observed with nonirritating scars after spinal fusion. Synovial fluid was aspirated with a needle under X-ray control to rule out a periprosthetic infection. No evidence of an infectious process (alpha defensin ELISA: 0.1; leukocyte esterase test negative; cell count 1678/*μ*l (33.3% polymorphonuclear). An appointment for the surgical operation was arranged with the patient to relieve the left hip joint on two forearms walking aids until revision surgery.

An anteroposterior X-ray of the pelvis was performed for the operation and the prosthetic components. A senior orthopedic surgeon with more than 10 years of experience in THA performed the operation in the right lateral position via a posterior approach to the hip. Intraoperatively, the abrasion granuloma was already fused with the fascia lata and encompassed the entire proximal and middle femur. The sciatic nerve has grown with the granuloma over almost 20 cm. After the joint had opened, copious amounts of black discolored synovial fluid were emptied. The entire joint presented itself as metallotic.

**Figure 3:**
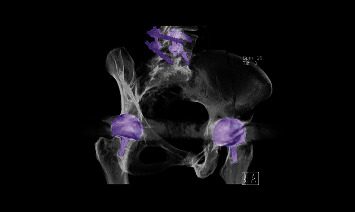
CT volume rendering of the pelvis. The reconstruction of all CT planes gives an overview of the fracture of the Os ilium and the extent of the cyst.

**Figure 4:**
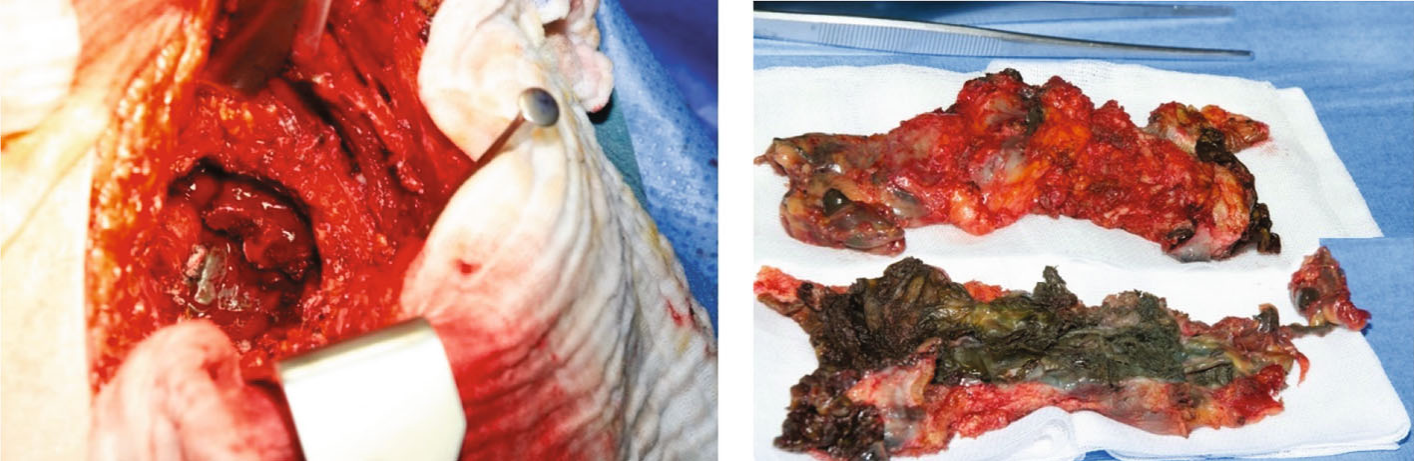
Metallotic pseudotumor. (a) shows the intraoperative metallosis with pronounced osteolysis. (b) shows the resected metallotic tissue after a complete surgical debridement.

Pronounced osteolysis is observed in the femoral neck. The anterior edge of the cup was no longer visualized for osteolytic reasons. The ilium, the pubis, and the sciatic bone were lined with pronounced osteolytic linings (Figure 3). Surprisingly, in the case of osteolytic ilium fracture, no relative movement of the ileum is observed, showing that the pelvis osteosynthesis treatment could be dispensed. The acetabulum was debrided, and the metallosis was shown here at the smaller pelvis, carefully and extensively debrided (Figure 4).

**Figure 5:**
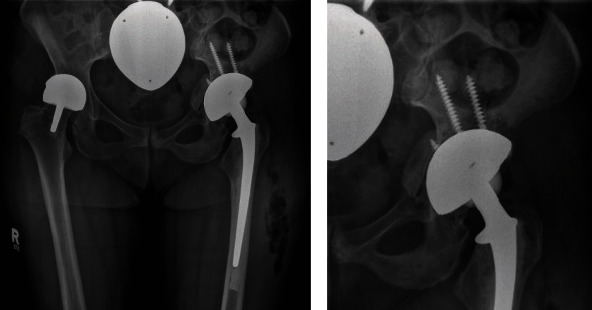
Surgical reconstruction after implantation of a cemented THA. (a) is an anteroposterior X-ray of the pelvis that shows the postoperative situation. On the left side, the patient carries a McMinn-like prosthesis without any symptoms or functional deficits. In (b), the bony acetabular reconstruction is observed. The cup was fixated with three screws to increase rotatory stability.

Two femoral heads from the inhouse bone bank were prepared for the construction. Two larger bone lids were shaped as a pan-base plastic, partially replaced the anterior pillar, and splinted in the acetabulum. The larger defects were filled with the remaining bone, resulted in a relatively stable bony bed. Then, a Trabecular Metal Acetabular Revision Shell (Zimmer Biomet, Warsaw, IN, USA) with an outer diameter of 60 mm was inserted, screwed into the dorsal of the abutment with 4 screws. An Endo-Mark III cup (Waldemar Link GmbH, Hamburg, Germany) 49/32.5 mm was cemented into this. Carried out is the femoral implantation of a Lubinus Classic Plus shaft (Waldemar Link GmbH, Hamburg, Germany), size 4, 126° CCD angle, 150 mm long (Figure 5).

The patient was mobilized postoperatively with physiotherapeutic exercise treatment. Postoperatively, a partial 10 kg weight bearing for 6 weeks was recommended. After 6 weeks, the patient is advised to increase the load by 10 kg/week until the full load is reached. To prevent dislocation, strict avoidance of internal rotation, adduction, and flexion over 90 degrees was recommended for 3 months postoperatively. Thrombosis prophylaxis with enoxaparin 40 mg a day was carried out until the patient was fully stressed. After an inconspicuous inpatient stay, the patient was discharged on the sixth postoperative day. Six weeks after the operation, the patient began a 3-week follow-up treatment.

**Figure 6:**
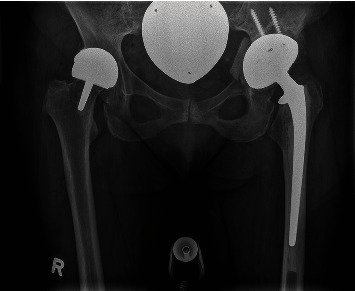
Follow-up X-ray 6 months after surgery. The anteroposterior view of the pelvis shows the correct placement of the implants and a boney healing of the Os ilium.

In the follow-up examination 6 months after surgery, the patient mobilized herself freely and with full weight bearing on the left leg (Figure 6). Function of the left hip was within physiological range.

## 3. Discussion

Hip resurfacing is attractive for reducing the risk of femoral head dislocation and is effective for younger patients with primary osteoarthritis, good bone quality, and high demands of sports activity. The results of this study show significantly worse outcomes in female patients, patients > 50 years, patients with small femoral heads (<50 mm), when the cup inclination is greater than 55 degrees, and in patients with congenital or acquired bone diseases, such as osteoporosis, femoral head necrosis, or hip dysplasia [3, 8].

In this case, several factors are responsible for the poor performance of the prosthesis. The patient was already 64 years old when the primary hip resurfacing implantation was performed. Women have significantly worse outcomes after hip resurfacing, according to the status. The Australian National Joint Replacement (NJR) Registry showed revision rates of 19.1% after 10 years in women < 55 years. However, revision rates in men of the same age were only 6.5% after 10 years [9]. It is unclear whether the higher revision rates in women were associated with women more likely to have congenital hip dysplasia, face a higher risk of osteoporosis, and tend to have smaller femoral heads than men. Furthermore, a relatively small femoral head size of 42 mm diameter was implanted. The Australian NJR showed surgical revisions after 10 years in 17.6% of patients with femoral head sizes < 50 mm. However, in femoral head sizes > 50 mm, the case was 6.0%, which might be associated with the poorer distribution of synovial fluid between articulating components in small head sizes [9]. Furthermore, the anchorage of the femoral peg is significantly more stable with larger femoral heads since it is surrounded by more bone. However, the peg size remains the same in smaller femoral head sizes [10]. The patient's primary treatment was based on developed hip dysplasia, associated with poorer outcomes. In this case, lack of cranial acetabular roofing often complicates the correct setting of the inclination by approximately 45°, which leads to higher edge loading, increased dry friction, and progressive metal wear, resulting in premature failure [11]. In our case, the primary implantation of the acetabular component is regular at 47°, but with deep acetabular positioning. A study shows that even patients with well-positioned hip resurfacing can still experience metal wear and pseudotumors [7].

Metal ions, such as cobalt and chromium, are released during metal abrasion, leading to oxidative stress via various cell signals and stimulating the secretion of proinflammatory cytokines and indicating osteolysis via osteoclast activation [12, 13]. Normal blood concentrations of chromium and cobalt ions are 0.5 *μ*g/l and 0.8 *μ*g/l, respectively, often elevated in patients with chromium-cobalt alloys [14]. An increase in cobalt serum concentrations is a reliable indicator of abnormal metal wear [15–18]. However, a correlation between increasing metal ion concentrations and worsening renal function could not be demonstrated [19].

Matuszak et al. reported on 541 patients with hip resurfacing that it was shown that 12.2% had osteolytic lesions in the prosthesis after 2 years. High chromium blood concentrations and a steep inclination are independent predictors for osteolysis development or progression [20]. In another retrospective study of 102 patients with metal-on-metal hip resurfacing, cystic changes in bone were detected on MRI in 34% after 13 years of follow-up [21]. A total of 84% of these were either asymptomatic or had minimal symptoms. This “silent soft tissue pathology” is vital for young patients, as osteolytic bone changes can significantly complicate further replacement surgery [16]. In our case, the patient showed a clear progression of osteolysis in the acetabular region and the femoral neck with increasing migration of the acetabular component since 2019. The patient had no complaints at that time.

In some previous studies, we found many cases of periprosthetic fractures of the femoral neck after hip resurfacing [22–24]. Over the years, few cases of stress fractures or osteolytic leading to fractures of the Os pubis have been published [25]. In 5000 patients examined after hip resurfacing, the causes for revision at the femoral region were found in 56.6% of the cases (femoral neck fracture: 29.7%; femoral head necrosis: 16.5%; loosening of the femoral component: 10.4%). In comparison, acetabular loosening was the cause in only 17.6% of the cases [22]. In 2019, Joseph et al. presented a case of a 57-year-old female patient who underwent left hip resurfacing. The patient fell on the left side 12 years after primary implantation, resulting in an acetabular fracture with significant posterior column dislocation. Due to the severe dislocation, the patient underwent internal fixation. However, a prosthesis change was not performed despite existing osteolysis in the acetabular roof and femoral neck. The authors described the case as unique [26]. Novel approach and fixation are crucial for this type of fracture. However, the posterior approach is best suited for revision surgery of this case. For the treatment of periprosthetic fractures of the acetabular component, various cups, cages, and different tantalum augments are available, already achieved good results for acetabular defects [27].

After an extensive review of the literature, no report is found that focuses on a fracture of the ileum after hip resurfacing. Our case report shows that the wrong indication for hip resurfacing can have dramatic consequences for the patient. The patient already faces several risk factors, such as gender, age, femoral head size, and primary care for developed dysplasia of the hip. Therefore, hip resurfacing should be considered critically and be performed in exceptional cases.

## 4. Conclusion

We present a case report of osteoarthritis treated with hip resurfacing owing to the potential risks discussed. With good surgical care and prompt postoperative mobilization, the results after total hip arthroplasty are more superior to other surface replacements in terms of longevity and patient satisfaction. Even though the present case is an exception, it carries some risks regarding a metal-on-metal bearing couple. Since this case is unique in the literature, no guidelines are noted for surgical treatment. In our opinion, a periprosthetic fracture of this type should be performed in a hospital using extensive endoprosthetic expertise.

## Data Availability

The data used to support the findings of this study are available from the corresponding author upon request.
